# CNS microRNA profiles: a database for cell type enriched microRNA expression across the mouse central nervous system

**DOI:** 10.1038/s41598-020-61307-5

**Published:** 2020-03-18

**Authors:** Nathan Pomper, Yating Liu, Mariah L. Hoye, Joseph D. Dougherty, Timothy M. Miller

**Affiliations:** 1 Neurosciences Program, Division of Biology and Biomedical Sciences, Washington University School of Medicine in St. Louis, St. Louis, MO 63110 USA; 2 Department of Neurology, Washington University School of Medicine in St. Louis, St. Louis, MO 63110 USA; 3 Department of Genetics, Washington University School of Medicine in St. Louis, St. Louis, MO 63110 USA; 4 Department of Psychiatry, Washington University School of Medicine in St. Louis, St. Louis, MO 63110 USA

**Keywords:** Genetics of the nervous system, Molecular neuroscience

## Abstract

microRNAs are short, noncoding RNAs that can regulate hundreds of targets and thus shape the expression landscape of a cell. Similar to mRNA, they often exhibit cell type enriched expression and serve to reinforce cellular identity. In tissue with high cellular complexity, such as the central nervous system (CNS), it is difficult to attribute microRNA changes to a particular cell type. To facilitate interpretation of microRNA studies in these tissues, we used previously generated data to develop a publicly accessible and user-friendly database to enable exploration of cell type enriched microRNA expression. We provide illustrations of how this database can be utilized as a reference as well as for hypothesis generation. First, we suggest a putative role for miR-21 in the microglial spinal injury response. Second, we highlight data indicating that differential microRNA expression, specifically miR-326, may in part explain regional differences in inflammatory cells. Finally, we show that miR-383 expression is enriched in cortical glutamatergic neurons, suggesting a unique role in these cells. These examples illustrate the database’s utility in guiding research towards unstudied regulators in the CNS. This novel resource will aid future research into microRNA-based regulatory mechanisms responsible for cellular phenotypes within the CNS.

## Introduction

microRNAs (miRNAs) are short, regulatory RNA molecules that become functionally active after being incorporated into the RNA-induced silencing complex (RISC), containing Argonaute-2^1,2^. Canonically, miRNA bind target mRNA in their 3′ untranslated region via Watson-Crick base pairing to regulate translation by either direct translational inhibition or mRNA destabilization^3^. Because only partial complementarity is required for a miRNA to regulate mRNA translation, miRNAs can regulate hundreds of transcripts in a given cell type^4^. This widespread regulation enables miRNAs to profoundly shape the expression landscape and physiology of a cell. Similar to transcription factors, the cell type specific miRNA code can tune which genes are actively expressed in a given cell type^5^. In fact, several groups have demonstrated that ectopic miRNA expression is sufficient to direct and reprogram cell identity, including induced pluripotent stem cells and neurons^6,7^. It is therefore important to know which miRNAs are expressed in a given cell type, but this is difficult in a complex tissue such as the central nervous system (CNS).

Previous studies have performed miRNA-ome analyses of the CNS. For example, Bak and colleagues undertook a large scale analysis of miRNA expression across 13 distinct neuroanatomical regions, but lacked the resolution to describe miRNA expression on a cell type specific basis within these nervous system tissues^8^. To enable cell type specific resolution analysis of miRNA expression, He *et al*. developed a tool for genetic expression of a tagged Argonaute-2 in mice, characterizing miRNA expression in several neuronal cell types in cortex and cerebellum^9^. However, the field remains hindered by a lack of a thorough cell type specific analysis of miRNA expression across the CNS. Additionally, there has yet to be a publicly accessible and user-friendly database describing cell type specific miRNA expression within the nervous system.

To solve this second problem, we pooled data across studies utilizing this Cre-dependent miRNA affinity purification technique^9^ to identify cell type specific miRNAs across major cell types within the mouse CNS. In addition to the He study, we previously leveraged this affinity purification technique to identify miRNAs known to be important for particular cell types, such as the motor neuron-enriched miR-218^10–12^. This discovery enabled further study of how aberrant miR-218 expression in cell types that do not physiologically express miR-218, such as astrocytes, mediates their dysfunction in a disease like amyotrophic lateral sclerosis^13^. This example illustrates how identifying cell type specific expression of even a single microRNA can be the starting point for a productive line of experimental investigation.

In this report, we describe the creation of a website (miRNA.wustl.edu) to provide a platform for comparing miRNA expression for various cell types within nervous system tissue using data generated in Hoye *et al*., 2017^14^ and He *et al*., 2012^9^. Similar sources for protein-coding RNA, including the Brain RNA-Seq atlas^14^, have become widespread research tools in considering which cell types may be involved in an observed phenotype^15^. Our resource will assist future hypothesis generation and experimental analyses by informing on specific miRNA-mediated regulatory mechanisms in particular nervous system cell types.

## Results

We developed the database to allow interactions with the datasets in two ways – a ‘Search by miRNA’ function that will provide graphic illustration for expression of a given miRNA and related family members, and a ‘Search by Cell Type’ function that will allow the end user to conduct a variety of differential expression contrasts to identify miRNAs that may be enriched in a cell type of interest in an unbiased way. Below, we provide several vignettes demonstrating the utility of these functions.

### ‘Search by miRNA’ highlights the potential role of microglial miR-21a-5p in spinal cord injury

**Figure 1:**
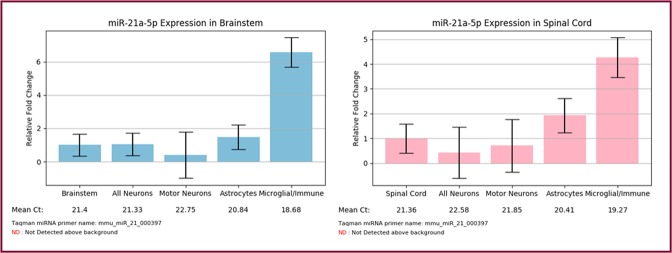
‘Search by miRNA’ output for miR-21. Expression is consistently enriched in microglial/immune cells within nervous system tissues under physiological conditions (n = 3–4 mice; mean ± SEM). The “Claret” colored frame indicates that miR-21a-5p is broadly conserved among most vertebrate, usually including zebrafish.

One common use for databases is to identify cell types of interest in a given phenotype. As a proof of principle, we leveraged our database to help generate a novel hypothesis regarding a previously undescribed role for microglial miRNA in spinal cord injury. Several groups have identified miR-21 as significantly upregulated after spinal cord injury, causing downregulation of targets FasL and PTEN, and its expression is directly related to secondary cell death following injury^16–18^. By *in situ hybridization*, miR-21 was expressed in neurons after injury but the majority of its expression seems to occur in other cell types^18^. One study demonstrated miR-21 expression in astrocytes following injury both promoted glial scar formation and hindered axonal regrowth, underscoring the importance of cell types beyond neurons in the miR-21 injury response^19^. Our database confirms the expression of miR-21 in spinal astrocytes but also indicates an even greater expression in spinal microglia, that is consistent with brainstem expression (Fig. 1). Therefore, for those interested in the importance of miR-21 after spinal cord injury, our database emphasizes that microglia likely play an underappreciated role. Consistent with this, some evidence directly points to the neurotoxic effect of microglial FasL and its repression by microglial miR-21^20^, but this has yet to be investigated in the context of spinal cord injury.

### ‘Search by miRNA’ reveals cell level regional variability in miR-326-3p expression

**Figure 2:**
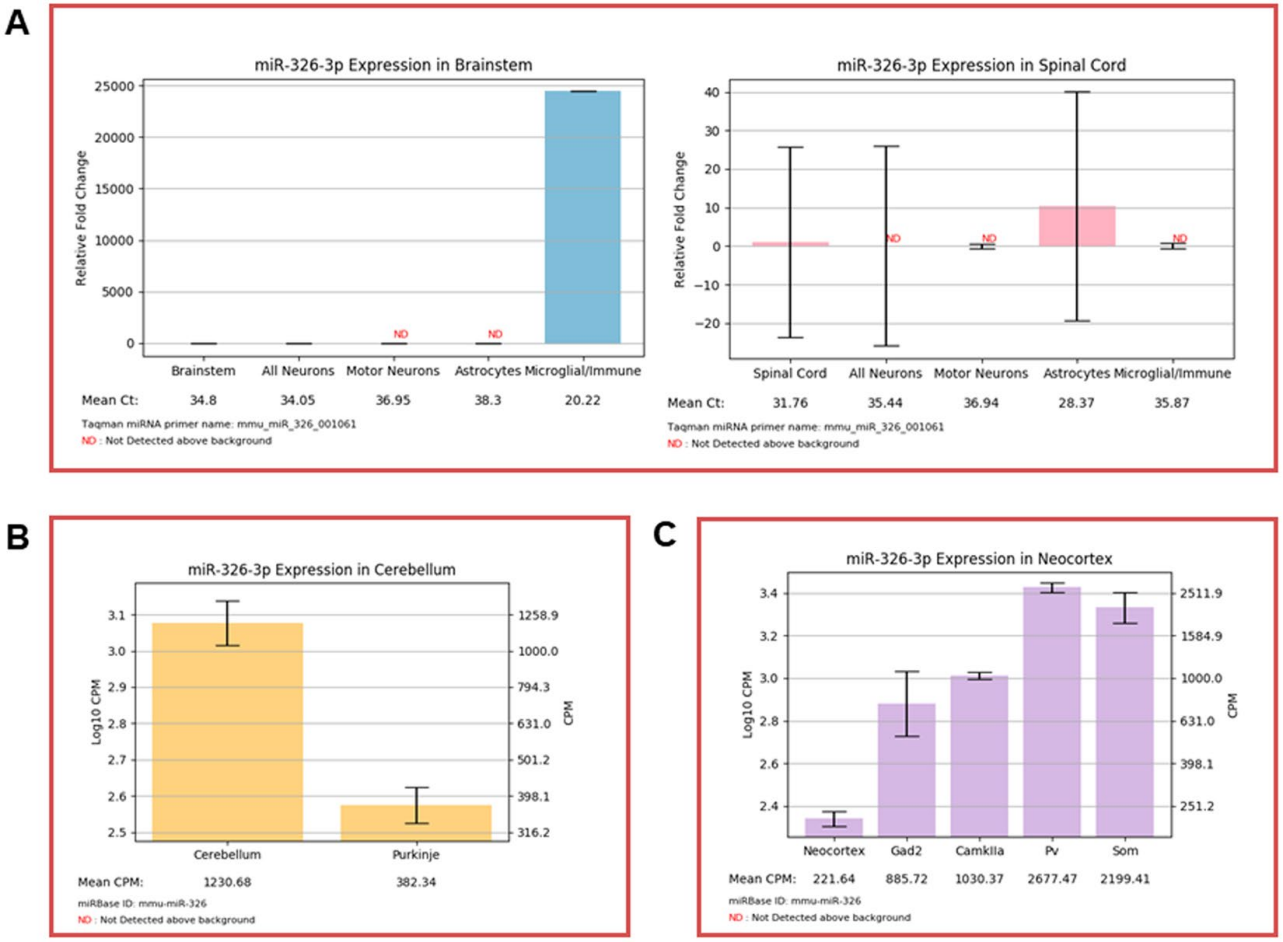
(**A**) ‘Search by miRNA’ output for miR-326-3p in the brainstem and spinal cord. Expression is readily detectable exclusively in microglia of the brainstem and astrocytes of the spine (n = 3–4 mice; mean ± SEM). **(B)** ‘Search by miRNA’ output for miR-326-3p in the cerebellum. miR-326-3p is depleted in Purkinje neurons, relative to the cerebellum as a whole (n = 2–3 mice; mean ± SEM). **(C)** ‘Search by miRNA’ output for miR-326-3p in the neocortex. miR-326-3p is readily detectable in cortical neurons (n = 2–3 mice; mean ± SEM). Only the more predominant and conserved 3p strand is displayed. The “Valencia” colored frame indicates that miR-326-3p is conserved among most mammals.

An additional use for our database is to compare regional expression of miRNAs, highlighting potential differences in development and physiology of cells within distinct locations. miR-326 is an extreme example of a potentially important regional difference. Our database identifies miR-326 as an almost exclusively microglial miRNA within the brainstem, however miR-326 is largely absent from spinal microglia. Within the spine, miR-326 expression is largely restricted to astrocytes (Fig. 2A). miR-326 has been implicated as a pro-inflammatory miRNA in circulating CD4+ T cells^21^, so this distinction may contribute to differences in immune and inflammatory responses within these neuroanatomically distinct structures. For example, in response to a common peroneal nerve injury, microglia are activated within the spine, but not the medulla^22^. Regional differences, such as those in miR-326 expression, could underlie this differential response. Interestingly, this regional difference is further complicated by extension to other nervous system regions. For example, miR-326 is enriched in the cerebellum over Purkinje neurons, consistent with previous findings that glial cells express substantially more miR-326 than neurons (Fig. 2B). However, unlike other assayed regions, miR-326 is readily detectable in neocortical neurons, particularly interneuron subtypes (Fig. 2C). This indicates further regional variability, where miR-326 is expressed by more rostral neurons despite the lack of substantial neuronal expression in the brainstem, spinal cord and cerebellum.

### miR-383-5p expression highlights a potential specific regulatory role in glutamatergic neurons in the cortex

**Figure 3:**
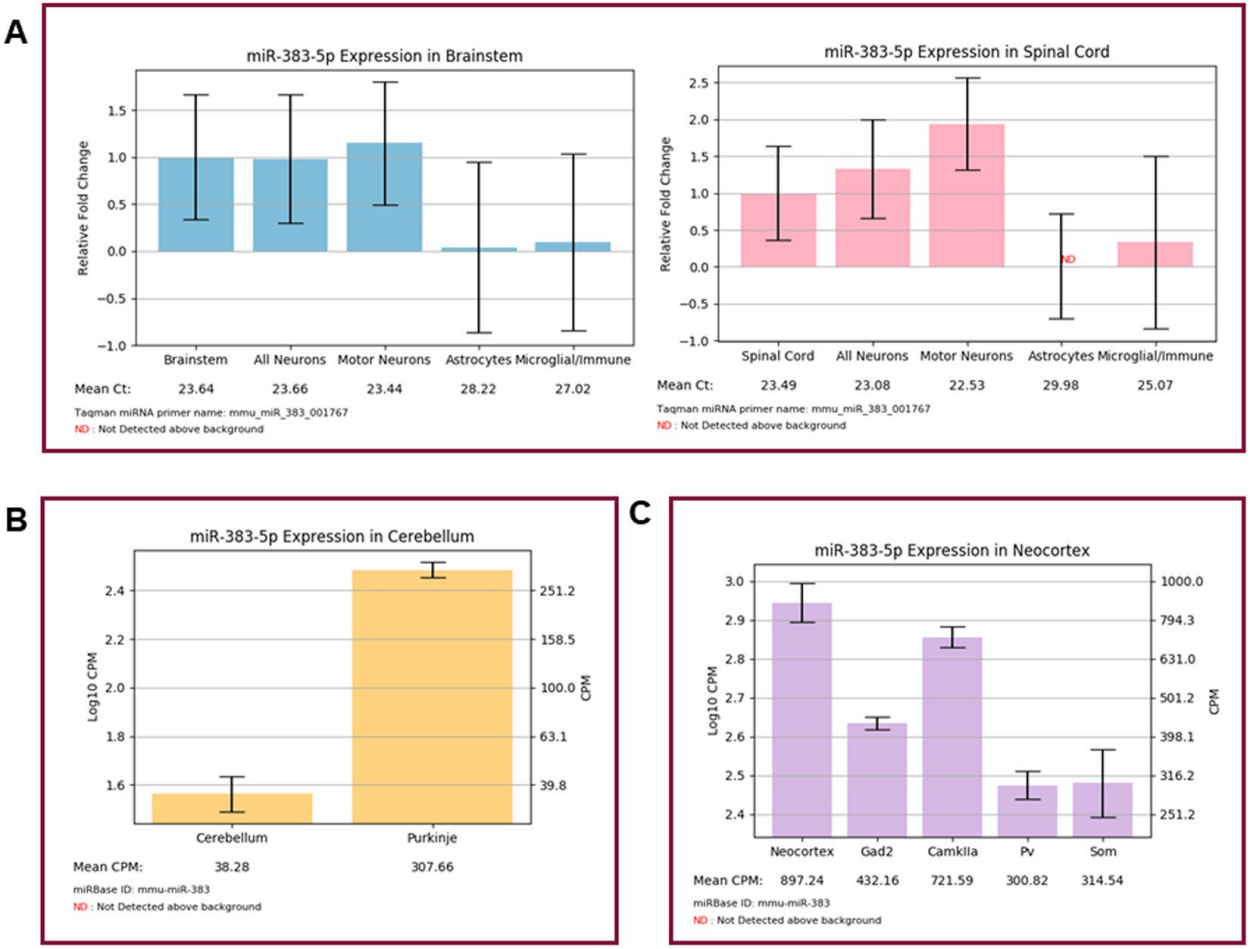
(**A**) ‘Search by miRNA’ output for miR-383-5p in the spine and brainstem. Expression is consistently and statistically enriched in neuronal cells over glia (n = 3–4 mice; mean ± SEM). **(B)** ‘Search by miRNA’ output for miR-383-5p in the cerebellum. Expression is enriched in Purkinje neurons, relative to the cerebellum as a whole (n = 2–3 mice; mean ± SEM). **(C)** ‘Search by miRNA’ output for miR-383-5p in the neocortex. Expression is highly expressed across neuronal subtypes but is most highly expressed by CamKIIa-positive glutamatergic neurons (n = 2–3 mice; mean ± SEM). Only the more predominant and conserved 5p strand is displayed. The “Claret” colored frame indicates that miR-383-5p is broadly conserved among most vertebrates.

Having two datasets included in our resource can provide complementary information. While Hoye *et al*. 2017 informs on neurons compared to glia, He *et al*. 2012 supplies information on neuronal subtypes. This is particularly informative in the case of a miRNA that is enriched in neurons over glia, but its neuronal subtype enrichment is less clear. miR-383-5p is an example of one such neuronal miRNA. In both the brainstem and spinal cord, miR-383 is enriched in all neurons, as well as motor neurons, compared to glial cells (Fig. 3A). miR-383 expression is similarly enriched in Purkinje neurons over the cerebellum as a whole, but this does not aid in identifying the particular neuronal subtypes that are responsible for the majority of miR-383 expression (Fig. 3B). However, the neocortical dataset reveals that miR-383 is not equally distributed across neuronal subtypes. miR-383 is particularly expressed by CamKIIa-positive glutamatergic neurons, relative to other neuronal subtypes (Fig. 3C). Interestingly, the expression of miR-383 has been tied to several neurodevelopmental and neuropsychiatric conditions. This includes Rett syndrome^23^, fragile X syndrome^24^, bipolar disorder^25^ and schizophrenia^26^. Further genomic-level analyses have revealed that the miR-383 locus and target genes are associated with an increased risk for autism spectrum disorder and linked behaviors, such as rumination^27,28^. These studies suggest that alterations in miR-383 levels and its corresponding targets may be detrimental to maintaining the proper function of neurons, particularly excitatory cortical neurons. It is also possible that miR-383 is mis-expressed by other neuronal subtypes. If supported by future experimental studies, such aberrant miR-383 expression could contribute to excitation-inhibition imbalance often associated with neurodevelopmental and psychiatric conditions.

### ‘Search by cell type’ identifies potential novel modulators of spinal motor neuron and immune cell function

**Figure 4:**
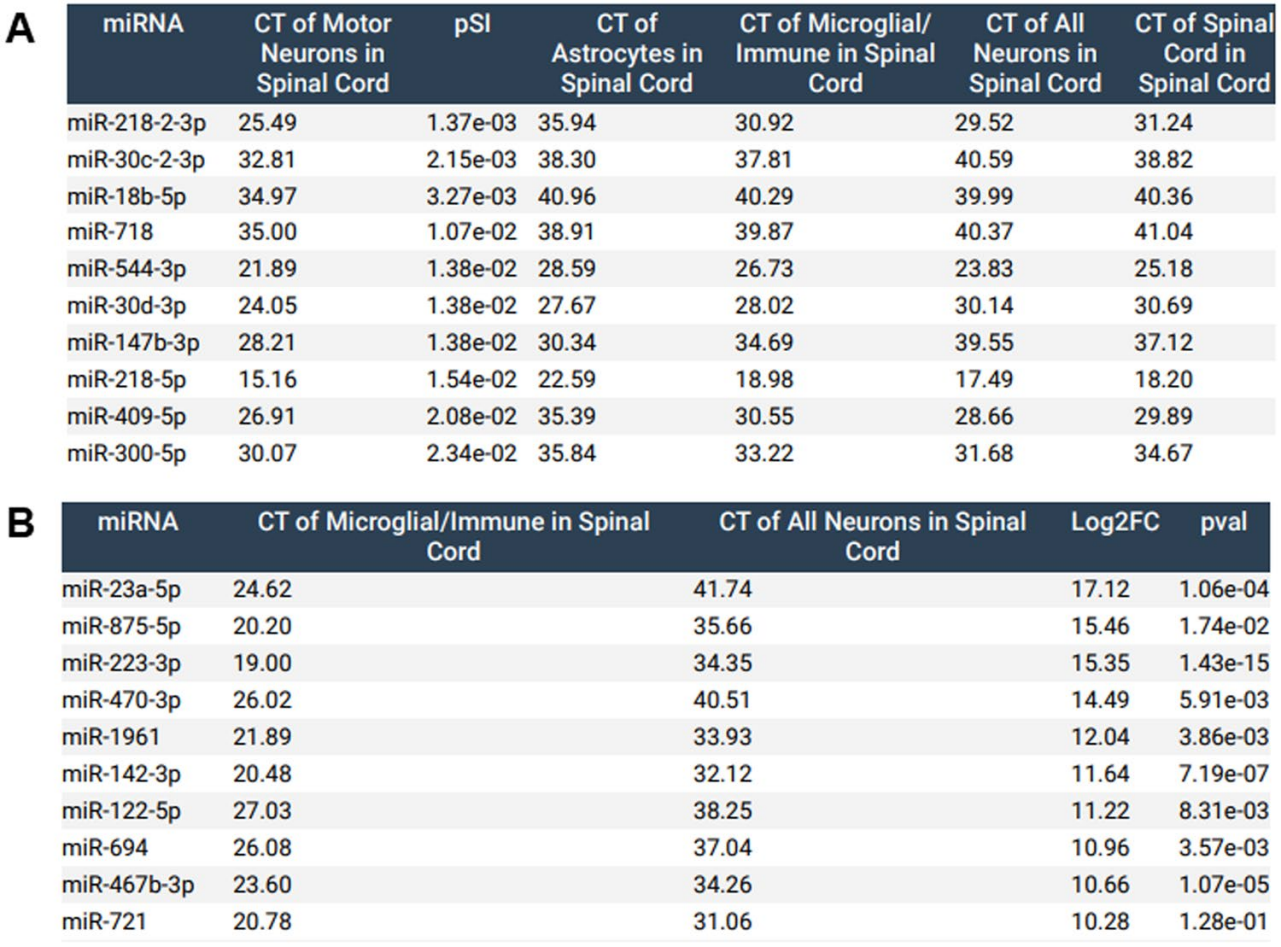
(**A**) Top 10 miRNAs of spinal motor neurons compared to all other cell types. miRNAs were selected and sorted by pSI value. **(B)** Top 10 microglial miRNAs as compared to all neurons in the spine, sorted by Log2FC.

We provide end-users with the functionality to search miRNA enrichment by cell type. This feature enables identification of the most abundant and/or most specifically expressed miRNA within a given cell type. For example, Fig. 4A details the top spinal motor neuron miRNA, relative to all other cell types assayed (including pan-neuronal miRNAs). This identifies both miR-218 strands, as well as potentially novel modulators of motor neuron function, including miR-544-3p. The loss of motor neuron-enriched miR-544 in whole spinal cord lysates has been associated with insults in the form of spinal cord injury^29^ or amyotrophic lateral sclerosis mutation-carrying muscle^30^, indicating a novel regulatory mechanism underlying motor neuron homeostasis. Additionally, Fig. 4B highlights the top 10 enriched miRNAs in spinal microglia as compared to all spinal neurons. This analysis yields miRNAs known to be associated with immune responses, including miR-223-3p^31^, but also novel miRNAs that have not been previously associated with microglial function. Overall, there were a number of miRNAs that were significantly expressed above background and relatively enriched in each cell type and CNS region (Table 1).

**Table 1:** Number of detectable miRNAs statistically enriched in each assayed population relative to tissue level expression.

	Brainstem	Spinal Cord	Neocortex	Cerebellum
All neurons	19	21	—	—
Motor neurons	52	60	—	—
Astrocytes	54	63	—	—
Microglia	59	57	—	—
Interneurons	—	—	102	—
Glutamatergic neurons	—	—	90	—
PV + interneurons	—	—	115	—
SOM + interneurons	—	—	152	—
Purkinje neurons	—	—	—	155

## Discussion

In this report, we detail the creation of a publicly accessible and user-friendly website for analyzing cell type enriched microRNA expression and demonstrate its utility to several distinct biological scenarios.

The dual ‘Search by miRNA’ and ‘Search by cell type’ functionality will enable end-users to quickly determine which neural cells may be associated with a given miRNA-associated phenotype they might observe throughout the course of their study. For example, we leveraged our database to identify microglia-enriched miR-21 regulation of FasL as a likely modulator to secondary neuron injury in spinal cord injury models. It can be equally informative to know which miRNAs are not expressed by a given physiological cell type. For example, the fact that adult astrocytes do not express miR-218, supports the hypothesis that motor neuron-derived miR-218 may contribute to the loss of astrocytic EAAT2 seen in amyotrophic lateral sclerosis^13^. Understanding which cell types express a particular miRNA may also guide development of inhibitors for miRNA, for example miR-155 in amyotrophic lateral sclerosis^32,33^.

One limitation of our database is that the data were produced under physiological conditions in healthy, adult mice. It will be important to incorporate additional information into the database based on cell type specific miRNA expression under a variety of conditions, such as across multiple developmental time points. Additionally, we recognize that a more comprehensive miRNA analysis of other cell types in the neocortex and cerebellum, as well as a deeper analysis of the brainstem and spinal cord samples, for example by small RNA sequencing, would provide additional information. The use of this sequencing technology indeed produced a greater number of enriched miRNAs per cell type in the He data over the microarray approach utilized by the Hoye data, perhaps due to the increased sensitivity to detect less abundant miRNA.

The database is set up such that it can easily accommodate the incorporation of additional cell-specific miRNA information. While the database is presently limited to the central nervous system, this strategy can be applied to any tissue with characterized Cre driver lines. For example, we have previously utilized this miRNA-tagging and affinity purification technique to characterize differences between CD11c^+^ dendritic cells of the spleen and CNS infiltrates during experimental autoimmune encephalitis^34^.

Existing miRNA tools are useful for target prediction, but fail to provide context as to whether a given miRNA and its putative target mRNA are co-expressed in the same cell type. By pairing this database with current resources for mRNA expression, future studies can uncover novel cell type specific gene regulatory patterns and if these patterns are pathologically disrupted. This database adds to the existing body of resources available for studying miRNAs, including target prediction software and broadens the wealth of information available to researchers studying miRNA regulation.

## Methods

### Animals

More details concerning the animals used in the generation of this database can be found in Hoye *et al*. 2017^10^ and He *et al*. 2012^9^. For Hoye *et al*. 2017, tissue was harvested from n = 3–4 per group at P63. For He *et al*. 2012, tissue was harvested from n = 2–3 animals per group at P56. All animals were maintained on a C57BL/6 background. The following Cre lines were crossed to the loxP-STOP-loxP-GFP-myc-Ago2 line: Syn (all neurons), ChAT (motor neurons), GFAP (astrocytes), Lyz2 (microglia), Gad2 (interneurons), CamKIIa (glutamatergic neurons), Pv (parvalbumin-positive interneurons), SST (somatostatin-positive interneurons) and L7 (Purkinje neurons of the cerebellum).

### Data collection and analysis

The data used to generate this online database were taken from Hoye *et al*. 2017^10^ and He *et al*. 2012^9^, where a more detailed description of the data generation method can be found. Briefly, cell type specific miRNAs within the mouse brainstem (pons and medulla), whole spinal cord, neocortex and cerebellum were identified using a Cre-dependent miRNA affinity purification technique using cell type specific expression of a GFP-tagged Ago2, followed by anti-myc capture. In Hoye *et al*. 2017, the bound miRNA samples were analyzed by microarray. Ct (cycle threshold) counts were LoessM normalized^35^ for each as a measure of the miRNA expression in each cell type. In He *et al*. 2012, the bound miRNA samples were analyzed by small RNA sequencing. The resulting counts, renormalized to library size (as counts per million – CPM) were used here. For each tissue, an Ago2 affinity purification was performed to obtain a tissue-wide miRNA profile. In Hoye *et al*. 2017, identical myc immunoprecipitation experiments were performed in non-transgenic animals to determine background miRNA expression associated with the immunoprecipitation. The relative expression data, log2 fold changes between any two given cell types and the associated adjusted p-values, were generated for all pairwise comparisons between cell types in each tissue. The specificity index (pSI) value for each miRNA in each cell type is used to define its enrichment in the corresponding cell type as compared to all other cell types and total tissue^36^. pSI cutoffs can be varied to define very stringent enrichment (e.g. 10E-5), or moderate enrichment (<0.05). A miRNA was considered detectable in the brainstem and spinal cord if its average Ct ≤ 35 and if 2 of 3 biological replicates exceeded the median background Ct. For the neocortex and cerebellum, where immunoprecipitation background data was not available, a miRNA was considered detectable if its CPM ≥ 1 in at least two biological replicates.

### Database creation

The CNS microRNA profiles website was built with Django framework version 1.11 (https://www.djangoproject.com/weblog/2009/), using Python 3.6, SQLite database, and is deployed using mod_wsgi-express (https://github.com/GrahamDumpleton/mod_wsgi).

### Search by miRNA

The ‘Search by miRNA’ functionality allows a user to visualize the relative expression of a miRNA in the cell types within the brainstem, spinal cord, neocortex and cerebellum. Upon entering a miRNA name, the webpage will display a list of expression bar plots for the query miRNA and other miRNAs belonging to the same group. The top color frame contains the expression plots of the query miRNA in the available tissues. If the miRNA is not available in neocortex and cerebellum, the color frame will only contains its expression plots in brainstem and spinal cord, and vice versa. The expression plots in brainstem and spinal cord show the relative fold changes of the query miRNA in four cell types in brainstem and spinal cord, respectively. Within each bar graph, miRNA expression is normalized to all neurons ($$baseline=mean\,Ct\,of\,all\,neurons)$$, the relative fold change of which is set to 1.0. All other cell types are displayed as expression relative to all neurons ($${2}^{baseline-meanCt})$$. Mean Ct of each cell type, as a more direct measure of abundance, is shown beneath the cell type names on the x-axis. For RNAseq-based data, the y-axis instead represents log CPM. A second y-axis was added on the right side with the corresponding non-log CPM values shown. The error bars show the standard error of the mean. If the miRNA is not detectable in a cell type, an “ND” marker in red will be displayed. As the data for this database was generated in mice, we included conservation information for potential extension to human systems and because the more conserved miRNA are likely to be more predominantly expressed. This information is conveyed on the webpage via a colored frame around the miRNA of interest that represents its level of conservation. The conservation data was drawn from the TargetScan^37^ “miR Family” data table (http://www.targetscan.org/mmu_72/mmu_72_data_download/miR_Family_Info.txt.zip). Any figure can be downloaded as PDF or PNG images for use with attribution.

### Search by cell type

The ‘Search by cell type’ functionality allows a user to find miRNAs that are enriched in one cell type (cell type 1) compared to another cell type or all other cell types (cell type 2), in any one of the four tissues: brainstem, spinal cord, neocortex and cerebellum. After specifying the tissue type, cell type 1, cell type 2, sorting method, and number of top enriched miRNAs, the webpage will display a data table showing the top enriched miRNAs in cell type 1 relative to cell type 2, and its corresponding Ct / CPM value in each cell type. The miRNAs will be sorted by log_2_ fold change in descending order or p-value in ascending order, if directly comparing two cell types. The miRNAs will be sorted by pSI in ascending order, if comparing one cell type to all other cell types. The results table can be downloaded as a CSV data file, PDF, or Excel spreadsheet.

## Data Availability

An Excel spreadsheet containing the raw data from each dataset can be downloaded from the “Download Data” page on the website.
